# 2-(4-Methyl­phen­yl)-1*H*-imidazo[4,5-*f*][1,10]phenanthroline

**DOI:** 10.1107/S1600536811008853

**Published:** 2011-03-12

**Authors:** Ling Ma, Liping Lu, Miaoli Zhu

**Affiliations:** a Institute of Molecular Science, Key Laboratory of Chemical Biology and Molecular, Engineering of the Education Ministry, Shanxi University, Taiyuan, Shanxi 030006, People’s Republic of China

## Abstract

In the title compound, C_20_H_14_N_4_, all the non-H atoms are roughly coplanar with an r.m.s. deviation of 0.0776 Å. In the crystal, mol­ecules are linked by N—H⋯N hydrogen bonds, forming chains along the (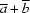). The chains are connected by inter­molecular C—H⋯N hydrogen bonds and π–π stacking inter­actions between inversion-related phenanthroline, imidazole and phenyl rings with centroid–centroid distances in the range 3.777 (1)–3.905 (1) Å.

## Related literature

For the biological activity of complexes of metal ions with 1,10-phenanthroline and its derivatives, see: Gao *et al.* (2009 ▶); Lu *et al.* (2003 ▶); Yuan *et al.* (2009 ▶, 2010 ▶); Chen *et al.* (2010 ▶). For aromatic π–π stacking inter­actions in related structures, see: Lu *et al.* (2004*a* ▶,*b* ▶,*c* ▶,*d* ▶); Ma *et al.* (2010 ▶); Ye *et al.* (2005 ▶); Zhang *et al.* (2005 ▶).
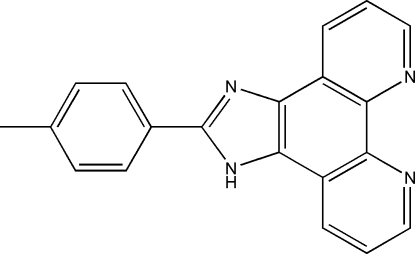


## Experimental

### 

#### Crystal data

C_20_H_14_N_4_
*M* *_r_* = 310.35
Monoclinic, 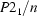

*a* = 9.1609 (8) Å
*b* = 15.5398 (13) Å
*c* = 11.725 (1) Å
β = 108.892 (1)°
*V* = 1579.2 (2) Å^3^
*Z* = 4
Mo *K*α radiation
μ = 0.08 mm^−1^
*T* = 298 K
0.30 × 0.20 × 0.20 mm

#### Data collection

Bruker SMART 1K CCD area-detector diffractometer
Absorption correction: multi-scan (*SADABS*; Sheldrick, 2000 ▶) *T*_min_ = 0.976, *T*_max_ = 0.984
10917 measured reflections
2790 independent reflections
2182 reflections with *I* > 2σ(*I*)
*R*_int_ = 0.021

#### Refinement

*R*[*F* ^2^ > 2σ(*F* ^2^)] = 0.037
*wR*(*F* ^2^) = 0.107
*S* = 1.05
2790 reflections
222 parameters
H atoms treated by a mixture of independent and constrained refinement
Δρ_max_ = 0.19 e Å^−3^
Δρ_min_ = −0.16 e Å^−3^

### 

Data collection: *SMART* (Bruker, 2000 ▶); cell refinement: *SAINT* (Bruker, 2000 ▶); data reduction: *SAINT*; program(s) used to solve structure: *SHELXS97* (Sheldrick, 2008 ▶); program(s) used to refine structure: *SHELXL97* (Sheldrick, 2008 ▶); molecular graphics: *ORTEP-3* (Farrugia, 1997 ▶) and *SHELXTL/PC* (Sheldrick, 2008 ▶); software used to prepare material for publication: *publCIF* (Westrip, 2010 ▶).

## Supplementary Material

Crystal structure: contains datablocks I, global. DOI: 10.1107/S1600536811008853/lw2058sup1.cif

Structure factors: contains datablocks I. DOI: 10.1107/S1600536811008853/lw2058Isup2.hkl

Additional supplementary materials:  crystallographic information; 3D view; checkCIF report

## Figures and Tables

**Table 1:** Hydrogen-bond geometry (Å, °)

*D*—H⋯*A*	*D*—H	H⋯*A*	*D*⋯*A*	*D*—H⋯*A*
N4—H4⋯N1^i^	0.908 (19)	2.106 (19)	3.0131 (19)	176.0 (17)
C1—H1⋯N3^ii^	0.93	2.57	3.479 (2)	165

Symmetry codes: (i) 
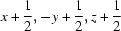
; (ii) 
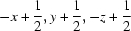
.

**Fig. 1.**
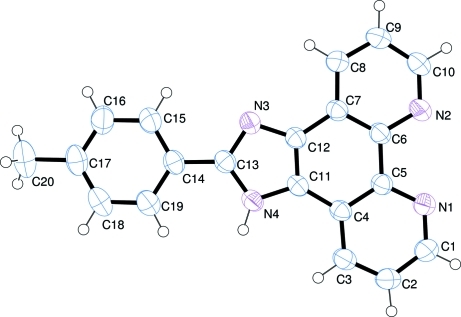
The views of the structures of (I) with displacement ellipsoids drawn at the 50% probability level.

**Fig. 2.**
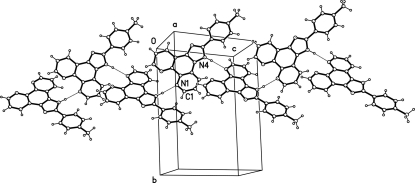
One-dimensional chain of (I) via N—H···N hydrogen bonds, dotted lines for hydrogen bonds.

## supplementary crystallographic information

### Comment

We have been interested in the bioactivity research of the complexes of metal ions with 1,10-phenanthroline and its derivatives, such as nuclease activity of mono (1,10-phenanthroline) copper complex (Lu *et al.*, 2003), protein tyrosine phosphatases inhibition activities of oxovanadium complexes with polypyridyl derivatives (Yuan *et al.*, 2009,2010; Gao *et al.*, 2009), selective OFF-ON fluorescent sensor for zinc in aqueous solution and living cells (Chen *et al.*, 2010). As we know, 1,10-phenanthroline and its derivatives, 2,2'-bipyridyl-like, are good planar ligands in metal-organic compounds, in which there are strong π–π stacking interactions (Ye *et al.*, 2005; Lu *et al.*, 2004*a*, 2004*b*, 2004*c*; 2004*d*; Zhang *et al.*, 2005; Ma *et al.*, 2010). We report here the structure of (I) (Fig. 1), which was synthesized from 1,10-Phenanthroline-5,6-dione and 4-methylbenzaldehyde. All non-hydrogen atoms of (I) are coplanar with a 0.0776 value of r.m.s. deviation of fitted atoms. The molecules are linked by N—H···N hydrogen bonds to form one-dimensional chains (Fig. 2), and the chains are connected by intermolecular C—H···N hydrogen bonds and π–π stacking interactions between inversion related phenanthroline and phenyl rings to complete the hydrogen bonding network in the crystal structure. The stacking distance π–π is in the range of 3.777 (1) to 3.905 (1) Å, namely *Cg*1···*Cg*3^i^ 3.905 (1), *Cg*2···*Cg*3^i^ 3.867 (1), *Cg*3···*Cg*3^ii^ 3.777 (1) Å. *Cg*1, *Cg*2, and *Cg*3 for the centroids of rings N1/C1—C5, C4—C7/C11/C12, C14—C19, respectively, symmetry codes i 1 - *x*,-*y*,1 - *z*; ii 2 - *x*,-*y*,1 - *z*.

### Experimental

1,10-Phenanthroline-5,6-dione (1.2 mmol), 4-methylbenzaldehyde (1.0 mmol), and ammonium acetate (4 mmol) were added in 20 ml of glacial acetic acid with a constant of stirring, and the mixture was refluxed for 2 h. When the reaction was cooled to room temperature, poured in 20 ml of water. The solution was neutralized with ammonia to pH 7. The precipitate was filtered off and recrystallized from methanol solution to give (I) for X-ray diffraction at room temperature.

### Refinement

H atoms attached to C atoms of (I) were placed in geometrically idealized positions and refined with *U*_iso_(H) = 1.2*U*_eq_(C) and *U*_iso_(H) = 1.5*U*_eq_(C), with C—H = 0.93 (aromatic) and 0.96 (CH_3_)Å. H atom attached to *N*(imidazole) in (I) was located from difference Fourier map and refined with N—H = 0.908 (19) (imidazole) Å.

### Crystal data

**Table:**

C_20_H_14_N_4_	*F*(000) = 648
*M**_r_* = 310.35	*D*_x_ = 1.305 Mg m^−^^3^
Monoclinic, *P*2_1_/*n*	Mo *K*α radiation, λ = 0.71073 Å
Hall symbol: -P 2yn	Cell parameters from 4474 reflections
*a* = 9.1609 (8) Å	θ = 2.5–27.4°
*b* = 15.5398 (13) Å	µ = 0.08 mm^−^^1^
*c* = 11.725 (1) Å	*T* = 298 K
β = 108.892 (1)°	Block, yellow
*V* = 1579.2 (2) Å^3^	0.30 × 0.20 × 0.20 mm
*Z* = 4	

### Data collection

**Table:**

Bruker SMART 1K CCD area-detector diffractometer	2790 independent reflections
Radiation source: fine-focus sealed tube	2182 reflections with *I* > 2σ(*I*)
graphite	*R*_int_ = 0.021
ω scans	θ_max_ = 25.0°, θ_min_ = 2.3°
Absorption correction: multi-scan (*SADABS*; Sheldrick, 2000)	*h* = −10→10
*T*_min_ = 0.976, *T*_max_ = 0.984	*k* = −18→18
10917 measured reflections	*l* = −13→13

### Refinement

**Table:**

Refinement on *F*^2^	Primary atom site location: structure-invariant direct methods
Least-squares matrix: full	Secondary atom site location: difference Fourier map
*R*[*F*^2^ > 2σ(*F*^2^)] = 0.037	Hydrogen site location: inferred from neighbouring sites
*wR*(*F*^2^) = 0.107	H atoms treated by a mixture of independent and constrained refinement
*S* = 1.05	*w* = 1/[σ^2^(*F*_o_^2^) + (0.047*P*)^2^ + 0.4106*P*] where *P* = (*F*_o_^2^ + 2*F*_c_^2^)/3
2790 reflections	(Δ/σ)_max_ = 0.001
222 parameters	Δρ_max_ = 0.19 e Å^−^^3^
0 restraints	Δρ_min_ = −0.16 e Å^−^^3^

### Special details

**Table:**

Geometry. All e.s.d.'s (except the e.s.d. in the dihedral angle between two l.s. planes) are estimated using the full covariance matrix. The cell e.s.d.'s are taken into account individually in the estimation of e.s.d.'s in distances, angles and torsion angles; correlations between e.s.d.'s in cell parameters are only used when they are defined by crystal symmetry. An approximate (isotropic) treatment of cell e.s.d.'s is used for estimating e.s.d.'s involving l.s. planes.
Refinement. Refinement of *F*^2^ against ALL reflections. The weighted *R*-factor *wR* and goodness of fit *S* are based on *F*^2^, conventional *R*-factors *R* are based on *F*, with *F* set to zero for negative *F*^2^. The threshold expression of *F*^2^ > σ(*F*^2^) is used only for calculating *R*-factors(gt) *etc*. and is not relevant to the choice of reflections for refinement. *R*-factors based on *F*^2^ are statistically about twice as large as those based on *F*, and *R*- factors based on ALL data will be even larger.

### Fractional atomic coordinates and isotropic or equivalent isotropic displacement parameters (Å^2^)

**Table:**

	*x*	*y*	*z*	*U*_iso_*/*U*_eq_	
C1	0.1494 (2)	0.34388 (11)	0.29392 (16)	0.0569 (5)	
H1	0.0796	0.3893	0.2775	0.068*	
C2	0.2314 (2)	0.32622 (12)	0.41361 (15)	0.0600 (5)	
H2	0.2142	0.3582	0.4751	0.072*	
C3	0.3377 (2)	0.26132 (11)	0.43997 (14)	0.0497 (4)	
H3	0.3958	0.2495	0.5195	0.060*	
C4	0.35793 (17)	0.21310 (9)	0.34572 (13)	0.0394 (4)	
C5	0.26563 (18)	0.23316 (10)	0.22634 (13)	0.0413 (4)	
C6	0.27420 (18)	0.18067 (10)	0.12531 (13)	0.0424 (4)	
C7	0.37132 (17)	0.10795 (10)	0.14623 (13)	0.0405 (4)	
C8	0.3685 (2)	0.05668 (11)	0.04748 (14)	0.0506 (4)	
H8	0.4290	0.0073	0.0587	0.061*	
C9	0.2767 (2)	0.07936 (12)	−0.06532 (15)	0.0607 (5)	
H9	0.2740	0.0464	−0.1321	0.073*	
C10	0.1872 (2)	0.15308 (12)	−0.07761 (15)	0.0635 (5)	
H10	0.1256	0.1686	−0.1548	0.076*	
C11	0.46288 (17)	0.14274 (10)	0.36043 (13)	0.0386 (4)	
C12	0.46786 (17)	0.09098 (10)	0.26710 (13)	0.0390 (4)	
C13	0.63601 (17)	0.04011 (10)	0.42481 (13)	0.0415 (4)	
C14	0.76079 (17)	−0.01205 (10)	0.50460 (13)	0.0433 (4)	
C15	0.8293 (2)	−0.07558 (12)	0.45554 (16)	0.0549 (5)	
H15	0.7935	−0.0854	0.3728	0.066*	
C16	0.9495 (2)	−0.12424 (13)	0.52785 (17)	0.0615 (5)	
H16	0.9942	−0.1658	0.4926	0.074*	
C17	1.0053 (2)	−0.11301 (13)	0.65087 (16)	0.0567 (5)	
C18	0.9361 (2)	−0.05052 (13)	0.69982 (16)	0.0600 (5)	
H18	0.9709	−0.0419	0.7828	0.072*	
C19	0.8162 (2)	−0.00045 (12)	0.62845 (15)	0.0538 (4)	
H19	0.7725	0.0414	0.6639	0.065*	
C20	1.1369 (2)	−0.16699 (15)	0.7291 (2)	0.0798 (7)	
H20A	1.2040	−0.1317	0.7914	0.120*	
H20B	1.1938	−0.1907	0.6806	0.120*	
H20C	1.0966	−0.2129	0.7649	0.120*	
N1	0.16445 (16)	0.29995 (9)	0.20174 (12)	0.0501 (4)	
N2	0.18375 (17)	0.20271 (9)	0.01305 (12)	0.0560 (4)	
N3	0.57503 (14)	0.02646 (8)	0.30708 (11)	0.0433 (3)	
N4	0.57054 (15)	0.10950 (8)	0.46160 (11)	0.0416 (3)	
H4	0.603 (2)	0.1350 (12)	0.5353 (17)	0.062 (5)*	

### Atomic displacement parameters (Å^2^)

**Table:**

	*U*^11^	*U*^22^	*U*^33^	*U*^12^	*U*^13^	*U*^23^
C1	0.0703 (12)	0.0485 (10)	0.0497 (10)	0.0146 (9)	0.0165 (9)	−0.0020 (8)
C2	0.0797 (13)	0.0563 (11)	0.0439 (10)	0.0147 (10)	0.0200 (9)	−0.0059 (8)
C3	0.0604 (10)	0.0491 (10)	0.0365 (8)	0.0031 (8)	0.0114 (7)	−0.0023 (7)
C4	0.0447 (8)	0.0365 (8)	0.0354 (8)	−0.0045 (7)	0.0107 (6)	−0.0015 (6)
C5	0.0469 (9)	0.0372 (8)	0.0377 (8)	−0.0005 (7)	0.0109 (7)	0.0004 (7)
C6	0.0481 (9)	0.0423 (9)	0.0338 (8)	0.0009 (7)	0.0089 (7)	0.0007 (7)
C7	0.0453 (9)	0.0412 (9)	0.0350 (8)	−0.0010 (7)	0.0130 (7)	0.0015 (6)
C8	0.0608 (10)	0.0507 (10)	0.0394 (9)	0.0100 (8)	0.0151 (8)	−0.0014 (7)
C9	0.0787 (13)	0.0637 (12)	0.0359 (9)	0.0129 (10)	0.0132 (8)	−0.0062 (8)
C10	0.0787 (13)	0.0678 (12)	0.0337 (9)	0.0174 (10)	0.0041 (8)	−0.0004 (8)
C11	0.0414 (8)	0.0388 (8)	0.0333 (8)	−0.0042 (7)	0.0089 (6)	0.0016 (6)
C12	0.0409 (8)	0.0391 (8)	0.0356 (8)	−0.0011 (7)	0.0104 (6)	0.0019 (6)
C13	0.0436 (8)	0.0417 (9)	0.0386 (8)	−0.0032 (7)	0.0127 (7)	0.0035 (7)
C14	0.0424 (8)	0.0460 (9)	0.0404 (8)	−0.0031 (7)	0.0120 (7)	0.0083 (7)
C15	0.0595 (11)	0.0585 (11)	0.0448 (9)	0.0087 (9)	0.0140 (8)	0.0092 (8)
C16	0.0611 (11)	0.0623 (12)	0.0620 (12)	0.0133 (9)	0.0213 (9)	0.0146 (9)
C17	0.0461 (9)	0.0642 (12)	0.0578 (11)	0.0003 (9)	0.0139 (8)	0.0233 (9)
C18	0.0540 (10)	0.0757 (13)	0.0432 (10)	−0.0034 (9)	0.0060 (8)	0.0141 (9)
C19	0.0533 (10)	0.0617 (11)	0.0443 (9)	0.0007 (8)	0.0126 (8)	0.0055 (8)
C20	0.0587 (12)	0.0927 (16)	0.0806 (15)	0.0122 (11)	0.0124 (11)	0.0390 (13)
N1	0.0613 (9)	0.0434 (8)	0.0418 (8)	0.0089 (7)	0.0114 (6)	0.0003 (6)
N2	0.0698 (10)	0.0544 (9)	0.0355 (7)	0.0137 (7)	0.0054 (7)	0.0003 (6)
N3	0.0457 (7)	0.0438 (8)	0.0379 (7)	0.0023 (6)	0.0099 (6)	0.0027 (6)
N4	0.0463 (7)	0.0417 (8)	0.0327 (7)	−0.0028 (6)	0.0070 (6)	−0.0001 (6)

### Geometric parameters (Å, °)

**Table:**

C1—N1	1.323 (2)	C11—C12	1.371 (2)
C1—C2	1.388 (2)	C11—N4	1.3744 (19)
C1—H1	0.9300	C12—N3	1.3751 (19)
C2—C3	1.366 (2)	C13—N3	1.3278 (19)
C2—H2	0.9300	C13—N4	1.370 (2)
C3—C4	1.396 (2)	C13—C14	1.465 (2)
C3—H3	0.9300	C14—C19	1.386 (2)
C4—C5	1.416 (2)	C14—C15	1.390 (2)
C4—C11	1.429 (2)	C15—C16	1.378 (2)
C5—N1	1.359 (2)	C15—H15	0.9300
C5—C6	1.461 (2)	C16—C17	1.376 (3)
C6—N2	1.3528 (19)	C16—H16	0.9300
C6—C7	1.410 (2)	C17—C18	1.382 (3)
C7—C8	1.399 (2)	C17—C20	1.511 (2)
C7—C12	1.431 (2)	C18—C19	1.384 (2)
C8—C9	1.364 (2)	C18—H18	0.9300
C8—H8	0.9300	C19—H19	0.9300
C9—C10	1.389 (3)	C20—H20A	0.9600
C9—H9	0.9300	C20—H20B	0.9600
C10—N2	1.322 (2)	C20—H20C	0.9600
C10—H10	0.9300	N4—H4	0.908 (19)
			
N1—C1—C2	123.95 (16)	N3—C12—C7	128.03 (13)
N1—C1—H1	118.0	N3—C13—N4	111.93 (13)
C2—C1—H1	118.0	N3—C13—C14	123.65 (14)
C3—C2—C1	119.18 (16)	N4—C13—C14	124.41 (14)
C3—C2—H2	120.4	C19—C14—C15	117.68 (15)
C1—C2—H2	120.4	C19—C14—C13	122.85 (16)
C2—C3—C4	118.99 (15)	C15—C14—C13	119.47 (14)
C2—C3—H3	120.5	C16—C15—C14	120.86 (17)
C4—C3—H3	120.5	C16—C15—H15	119.6
C3—C4—C5	118.32 (14)	C14—C15—H15	119.6
C3—C4—C11	124.86 (14)	C17—C16—C15	121.78 (19)
C5—C4—C11	116.79 (13)	C17—C16—H16	119.1
N1—C5—C4	121.73 (14)	C15—C16—H16	119.1
N1—C5—C6	117.80 (13)	C16—C17—C18	117.37 (16)
C4—C5—C6	120.46 (14)	C16—C17—C20	121.20 (19)
N2—C6—C7	121.69 (14)	C18—C17—C20	121.43 (18)
N2—C6—C5	118.08 (14)	C17—C18—C19	121.64 (17)
C7—C6—C5	120.20 (13)	C17—C18—H18	119.2
C8—C7—C6	118.11 (14)	C19—C18—H18	119.2
C8—C7—C12	123.68 (14)	C18—C19—C14	120.66 (18)
C6—C7—C12	118.22 (13)	C18—C19—H19	119.7
C9—C8—C7	119.71 (16)	C14—C19—H19	119.7
C9—C8—H8	120.1	C17—C20—H20A	109.5
C7—C8—H8	120.1	C17—C20—H20B	109.5
C8—C9—C10	118.15 (16)	H20A—C20—H20B	109.5
C8—C9—H9	120.9	C17—C20—H20C	109.5
C10—C9—H9	120.9	H20A—C20—H20C	109.5
N2—C10—C9	124.39 (16)	H20B—C20—H20C	109.5
N2—C10—H10	117.8	C1—N1—C5	117.73 (14)
C9—C10—H10	117.8	C10—N2—C6	117.91 (15)
C12—C11—N4	105.57 (13)	C13—N3—C12	104.57 (13)
C12—C11—C4	123.21 (13)	C13—N4—C11	106.80 (13)
N4—C11—C4	131.17 (14)	C13—N4—H4	127.0 (12)
C11—C12—N3	111.12 (13)	C11—N4—H4	125.3 (12)
C11—C12—C7	120.85 (14)		
			
N1—C1—C2—C3	1.8 (3)	C8—C7—C12—N3	2.6 (3)
C1—C2—C3—C4	−1.7 (3)	C6—C7—C12—N3	−177.08 (14)
C2—C3—C4—C5	−0.6 (2)	N3—C13—C14—C19	−175.68 (15)
C2—C3—C4—C11	−178.91 (16)	N4—C13—C14—C19	5.7 (2)
C3—C4—C5—N1	3.1 (2)	N3—C13—C14—C15	5.1 (2)
C11—C4—C5—N1	−178.52 (14)	N4—C13—C14—C15	−173.46 (15)
C3—C4—C5—C6	−175.51 (14)	C19—C14—C15—C16	−0.9 (3)
C11—C4—C5—C6	2.9 (2)	C13—C14—C15—C16	178.34 (16)
N1—C5—C6—N2	1.5 (2)	C14—C15—C16—C17	0.9 (3)
C4—C5—C6—N2	−179.89 (14)	C15—C16—C17—C18	−0.1 (3)
N1—C5—C6—C7	−176.74 (14)	C15—C16—C17—C20	179.85 (17)
C4—C5—C6—C7	1.9 (2)	C16—C17—C18—C19	−0.5 (3)
N2—C6—C7—C8	−2.1 (2)	C20—C17—C18—C19	179.49 (17)
C5—C6—C7—C8	176.11 (15)	C17—C18—C19—C14	0.5 (3)
N2—C6—C7—C12	177.60 (14)	C15—C14—C19—C18	0.3 (3)
C5—C6—C7—C12	−4.2 (2)	C13—C14—C19—C18	−178.97 (15)
C6—C7—C8—C9	1.9 (3)	C2—C1—N1—C5	0.6 (3)
C12—C7—C8—C9	−177.75 (16)	C4—C5—N1—C1	−3.0 (2)
C7—C8—C9—C10	−0.6 (3)	C6—C5—N1—C1	175.60 (15)
C8—C9—C10—N2	−0.6 (3)	C9—C10—N2—C6	0.5 (3)
C3—C4—C11—C12	172.70 (15)	C7—C6—N2—C10	0.9 (3)
C5—C4—C11—C12	−5.6 (2)	C5—C6—N2—C10	−177.34 (17)
C3—C4—C11—N4	−4.5 (3)	N4—C13—N3—C12	1.02 (17)
C5—C4—C11—N4	177.23 (15)	C14—C13—N3—C12	−177.72 (14)
N4—C11—C12—N3	0.13 (17)	C11—C12—N3—C13	−0.70 (17)
C4—C11—C12—N3	−177.65 (13)	C7—C12—N3—C13	178.22 (15)
N4—C11—C12—C7	−178.88 (13)	N3—C13—N4—C11	−0.97 (17)
C4—C11—C12—C7	3.3 (2)	C14—C13—N4—C11	177.76 (14)
C8—C7—C12—C11	−178.62 (15)	C12—C11—N4—C13	0.48 (16)
C6—C7—C12—C11	1.7 (2)	C4—C11—N4—C13	178.01 (15)

### Hydrogen-bond geometry (Å, °)

**Table:**

*D*—H···*A*	*D*—H	H···*A*	*D*···*A*	*D*—H···*A*
N4—H4···N1^i^	0.908 (19)	2.106 (19)	3.0131 (19)	176.0 (17)
C1—H1···N3^ii^	0.93	2.57	3.479 (2)	165

Symmetry codes: (i) *x*+1/2, −*y*+1/2, *z*+1/2; (ii) −*x*+1/2, *y*+1/2, −*z*+1/2.
